# UV-shielding properties of a cost-effective hybrid PMMA-based thin film coatings using TiO_2_ and ZnO nanoparticles: a comprehensive evaluation

**DOI:** 10.1038/s41598-023-34120-z

**Published:** 2023-05-02

**Authors:** Fatemeh Yousefi, Seyed Borhan Mousavi, Saeed Zeinali Heris, Samin Naghash-Hamed

**Affiliations:** 1 Faculty of Chemical and Petroleum Engineering, University of Tabriz, Tabriz, Iran; 2 J. Mike Walker ’66 Mechanical Engineering Department, Texas A&M University, College Station, TX 77843 USA; 3 Research Laboratory of Polymer, Department of Organic and Biochemistry, Faculty of Chemistry, University of Tabriz, Tabriz, Iran

**Keywords:** Engineering, Chemical engineering

## Abstract

This study aimed to assess the UV-shielding features of the PMMA-based thin film coatings with the addition of TiO_2_ and ZnO nanoparticles as nanofillers considering different contents. Furthermore, the effect of TiO_2_/ZnO nanohybrids at different ratios and concentrations was examined. The XRD, FTIR, SEM, and EDX analyses characterized the prepared films' functional groups, structure, and morphology. Meanwhile, the coatings' optical properties and UV-protecting capability were investigated by ultraviolet–visible (UV–Vis) spectroscopy. The UV–Vis spectroscopic study revealed that as the concentration of nanoparticles increased in the hybrid-coated PMMA, the absorption in the UVA region increased. Overall, it can be concluded that the optimal coatings for PMMA were 0.1 wt% TiO_2_, 0.1 wt% ZnO, and 0.025:0.025 wt% TiO_2_: ZnO nanohybrid. Considering the acquired FT-IR of PMMA with different content of nanoparticles before and after exposure to the UV irradiation, for some films, it was confirmed that the polymer-based thin films degraded after 720 h, with either decreasing or increasing intensity of the degraded polymer, peak shifting, and band broadening. Notably, the FTIR results were in good agreement with UV–Vis outcomes. In addition, XRD diffraction peaks demonstrated that the pure PMMA matrix and PMMA coating films did not show any characteristic peaks indicating the presence of nanoparticles. All diffraction patterns were similar with and without any nanoparticles. Therefore, it depicted the amorphous nature of polymer thin film.

## Introduction

Lately, polymer-based coatings with active interface layers have been used to protect against UV/IR waves by absorbing UV light and transmitting visible light. Hybrid polymer thin films have attracted noticeable consideration due to their excellent chemical, thermal, optical, and physical characteristics. Incorporating metal oxide nanoparticles into polymers promotes their mechanical and optical features^1–3^. The prepared polymeric waveguides need a little modification of the refractive index to perform total reflectance and influential waveguiding. Although using nanoparticles boosts polymers’ inherent properties, in some cases, they lead to low transparency; thus, choosing a functional nanoparticle is an essential factor^4^. Moreover, preparing a uniform dispersion of nanoparticles and polymers is still challenging^5–7^.

Typically, there are four main procedures for distributing nanoparticles in polymers: (1) preparation of a mixture of polymer and nanoparticles using extrusion for polymer melts at high shear rates; (2) evaporating the solvent after mixing the polymer/nanoparticles mixture at high shear rates or ultrasonic irradiation. It should be noted that uniform distribution cannot be fulfilled by only stirring the mixture without optimized interaction between the polymer matrix and nanoparticles and chemical bonding; (3) in-situ polymerization using nanoparticles; (4) chemical surface modification to graft a functional polymer molecule to the active sites on the nanoparticles’ surface. Previous investigations have confirmed that to prepare a homogenous polymer/nanoparticle dispersion, the content of nanoparticles must be kept at less than 10 wt% due to their high surface energy and immiscibility^8–10^.

Transparent plastic, such as poly(methyl methacrylate) (PMMA), is a common synthetic amorphous polymer broadly utilized in various applications, such as transportation, aerospace, electronics, and medical equipment, etc*.*, due to its remarkable characteristics, namely high transparency, abrasive and weather resistance, biocompatibility, cost-effectiveness, and good electrical insulation behavior. In addition, PMMA has excellent resistance to sunshine exposure since it shows a small variation under the influence of UV radiation, considerable thermal resilience in temperatures ranging from − 70 °C to 100 °C, and noticeable optical features with a refractive index of 1.490^11^. Protection of transparent plastic against UV-induced degradation is vital since exposure to UV light degrades this type of plastic. Zheng et al.^12^ prepared a novel PMMA-based coating and assessed its mechanical and optical attributes. Their findings showed that the prepared coating could maintain good optical properties, transparency, and visible light features. Additionally, the remarkable adhesion and hardness results revealed that this coating could pave the way for future design development of PMMA-based coatings. Zhang et al.^13^ successfully synthesized a novel PMMA-based coating using hyperbranched poly(urethane-phosphine oxide) and evaluated its optical features. They asserted that the prepared coating had excellent transparency as well as adhesion and fire safety. Sengwa et al.^14^ conducted a comprehensive examination toward structural, thermal, and optical characteristics of a prepared PMMA-based hybrid nanocomposites, proving that its structural property led to possession of great optical features, UV-blocker. Other studies have also shown the noticeable properties of PMMA-based coatings as a UV-shielding, anti-corrosion, and biocompatible material^15–17^.

Among different nanoparticles, high refractive index metal oxides, such as CeO_2_, TiO_2_, ZrO_2_, and ZnO have been widely used^18–20^. Titanium oxide (TiO_2_) is considered as a practical nanoparticle in surface protection applications since it has a wide energy band gap of 3 eV$\sim$, showing a strong UV light cutoff. Previously conducted works proved that thin films including TiO_2_ could effectively protect UV-degradable materials, especially PMMA^21,22^. Harb et al.^23^ successfully synthesized homogeneous PMMA-TiO_2_ and PMMA-ZrO_2_ nanocomposites and studied their protective properties. Their findings confirmed that PMMA-TiO_2_ nanocomposites showed excellent optical features due to their great biocompatibility, transparency, and thermal stability. Nagasawa et al.^24^ studied the optical properties of PMMA-TiO_2_ coatings prepared via the chemical vapor deposition method. The results exhibited that the prepared coatings had an excellent UV-protection performance, absorbing 99% of UV light in the wavelength range of 200–280 nm and with visible light transmittance of 95%.

Zinc oxide (ZnO) nanoparticles are a special multifunctional inorganic additive with remarkable features, including excellent UV-shield and antibacterial characteristics, large exciton binding energy of 60 $\sim$ meV, high refractive index, and high thermal conductivity^25–27^. ZnO nanoparticles demonstrate a near-band-edge (NBE) UV line with substantial visible luminescence. The efficient surface passivation and consistency are considered the most significant factor of the high luminescence efficiencies of ZnO nanoparticles. It has been reported that polymers containing ZnO nanoparticles had better surface passivation and lower surface-related visible emissions^8^. It was shown by Soumya et al.^19^ that using ZnO to prepare a hybrid PMMA-based coating via an in-situ polymerization method could effectively enhance NIR reflectance, photoluminescence, and UV-shielding characteristics. In a study conducted by Viorica et al.^28^, they prepared a hybrid ZnO/PMMA thin film coating to investigate its optical features. The outcomes exhibited that the hybrid coating had lower surface roughness and higher optical transmittance in Vis–NIR, 90%, compared to the pure sample, showing the effectiveness of incorporating ZnO into the polymer matrix.

Considering the comprehensive conducted literature review, most investigations in the stated area exhibited that the optical properties of PMMA-based thin film coatings can be further enhanced by incorporating nanofillers, especially TiO_2_ and ZnO nanoparticles; however, the effect of different content of nanofillers has not been considered. In addition, the effect of hybrid TiO_2_-ZnO nanofillers on the optical features of PMMA coatings has not been well understood. Furthermore, no studies have been paid to compare two different nanoparticles and their hybrid. Thus, in this study, for the first time, various content of TiO_2_, ZnO, and TiO_2_-ZnO nanohybrids were considered for preparing thin film PMMA coatings and scrutinizing their optical attributes. It is worth stating that although previous works have not focused on the industrial aspect of the ZnO/TiO_2_-PMMA thin film coatings, the outcomes of this study revealed that the prepared samples could be used for industrial purposes due to their remarkable UV-shielding features, cost-effectiveness, and environmentally friendly.

In this experimental study, TiO_2_-PMMA, ZnO-PMMA, and nanohybrid TiO_2_/ZnO-PMMA thin film coatings were successfully prepared at different content of nanoparticles, 0.05, 0.1, and 0.2 wt% to assess the UV-shielding properties of the prepared samples. The SEM, EDX, FT-IR, XRD, and UV–Vis techniques were employed to study the samples’ and nanoparticles’ morphological, structural, and thermal properties, and the UV-shielding capability of the coatings.

## Experimental

### Materials

Transparent PMMA granules as a base polymer were purchased from Xiamen Keyuan Plastic Co, China, ZnO and TiO_2_ nanoparticles as nanofillers were commercially purchased from VCN Materials, Iran. Tetrahydrofuran (THF) as a solvent was obtained from Merck, Germany. The main characteristics of the nanoparticles and PMMA from the manufacturer's data are listed in Tables 1 and 2, respectively. The structure morphology of prepared specimens was analyzed via scanning electron microscopy (SEM, Tescan Mira3 FEG-SEM, Czech). The element distributions in the surface of the prepared samples were also measured using X energy dispersive X-ray (EDX). The thorough study of the functional groups and degradation rate of pure PMMA and prepared nanohybrid coatings was recorded by Fourier transform infrared spectroscopy (Brucker Tensor 27, Germany). To determine the crystallite structure of nanoparticles, X-ray diffraction (XRD, Philips PW1730, Netherlands) (Cu-Kα (λ = 1.54056 A°) radiation at a voltage of 40 kV and 30 mA was applied. The XRD patterns were scanned over the angular radius of 2θ = 20–80° with a step size of 0.05°/s. The thermal resistance and degradation of the modified polymer using a UV lamp were assessed employing the thermo-gravimetric analysis via Linseis L81A1750, USA TGA analyzer. The amount of transmitted light in the visible region, which indicates the transparency of the films, and the amount of light absorption in the ultraviolet region, which determines the amount of light absorption that destroys the films, were investigated using UV–visible absorption spectra (UV–Vis, Analytic Jena Specord 250, UK).

**Table 1:** Physical characteristics of nanoparticles.

Nanoparticles	Purity (%)	Size (nm)	Density (g/cm^3^)	Shape and color
TiO_2_	99.9	30–50	4.23	Anatase, white
ZnO	99.8	10–30	5.60	Spherical, white

**Table 2:** Properties of PMMA.

Properties	Unit	ASTM standard	Value
Density	g/cm^3^	D-792	1.19
Melt flow rate	g/min	D-1238	1.48
Tensile strength	kg/cm^2^	D-638	10,200
Flexural strength	kg/cm^2^	D-790	15,600
Elongation at break	–	D-638	5%
Izod notched impact strength	kJ/m^2^	D-256	> 25
Heat deflection temperature	°C	D-648	> 100

### Coatings preparation

In this work, granulated poly(methyl methacrylate) (PMMA) polymer as the base polymer and TiO_2_ and ZnO nanoparticles in different percentages, 0.05, 0.1, and 0.2 wt%, as UV absorbers to improve the properties of the base polymer was used. The effect of TiO_2_ and ZnO nanoparticles and TiO_2_–ZnO (1:1) nanohybrid was also studied on the prepared coatings' optical properties.

**Figure 1:**
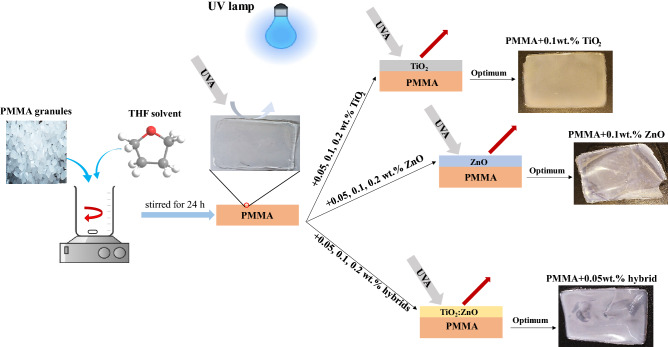
Procedure of coating preparation.

To prepare the composite film, 10 g of granulated PMMA was dissolved in 100 g of THF solvent. For this purpose, 100 g of THF was placed on a magnetic stirrer; then, the granules were slowly added to the solvent in order to prevent the granules from sticking. The solution was stirred for 24 h at room temperature. The homogeneous solution was placed in a Pyrex container and dried. The nanocomposite films were prepared by adding the additives to the solvent and putting them inside an ultrasonic bath (Parsonic 2600 s, Iran) for 5 h to achieve a uniform solution. Then, the granules were slowly added to the prepared solution and dried. Since the thickness of the prepared films and the drying process could affect the FT-IR, UV–Vis, TGA, and XRD results, the environmental conditions, including the temperature, and the thickness of the samples after drying, must be the same. Accordingly, all samples were placed at room temperature and covered with aluminum foil to prevent dust and other pollutants in the air from settling on the solution. Also, in order to provide an environment in which solvent vapors can escape, aluminum foil was perforated to prevent these vapors from being trapped inside the container. Moreover, using the same container for drying led to achieving the same thickness of samples. Finally, the prepared samples were in contact with a UV lamp for 720 h to study the degradation effect. The procedure of coating preparation is summarized in Fig. 1.

## Results and discussion

### UV–Vis analyses

The main objective of this work is to synthesize a coating with the property of absorbing light in ultraviolet wavelengths and at the same time, maintaining its transparency. For this reason, to determine the right composition, firstly, the property of absorbing ultraviolet light with high transparency was checked. In UV–Vis analysis, pure coating film (without nanoparticles) were used as a reference film to check the changes and evaluate the effect of additives compared to it^29^.

**Figure 2:**
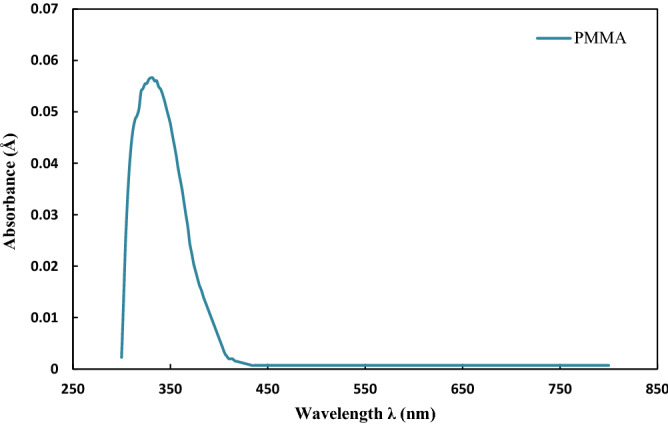
UV–Vis spectrum of PMMA.

In the first stage, the degradation of pure PMMA polymer were investigated in the presence of a UV lamp (Fig. 2). Considering Fig. 2, the top of the absorbance peak was seen at λ_max_ = 330 nm, confirming the degradation of the polymer coating at this wavelength. The UV-absorbance in the UV–Vis spectroscopy occurs due to electrons’ promotion to a higher energy state, which is described as electron transitions. The observed absorption trend in the case of PMMA can be justified by π → π* and n → π* transitions of electrons attributed to the C=O group in the ester functional group of PMMA^30,31^.

In the following, samples containing TiO_2_, ZnO, and TiO_2_/ZnO nanoparticles were investigated in two states, before and after, being exposed to ultraviolet rays.

#### PMMA + TiO_2_ nanoparticles coating

**Figure 3:**
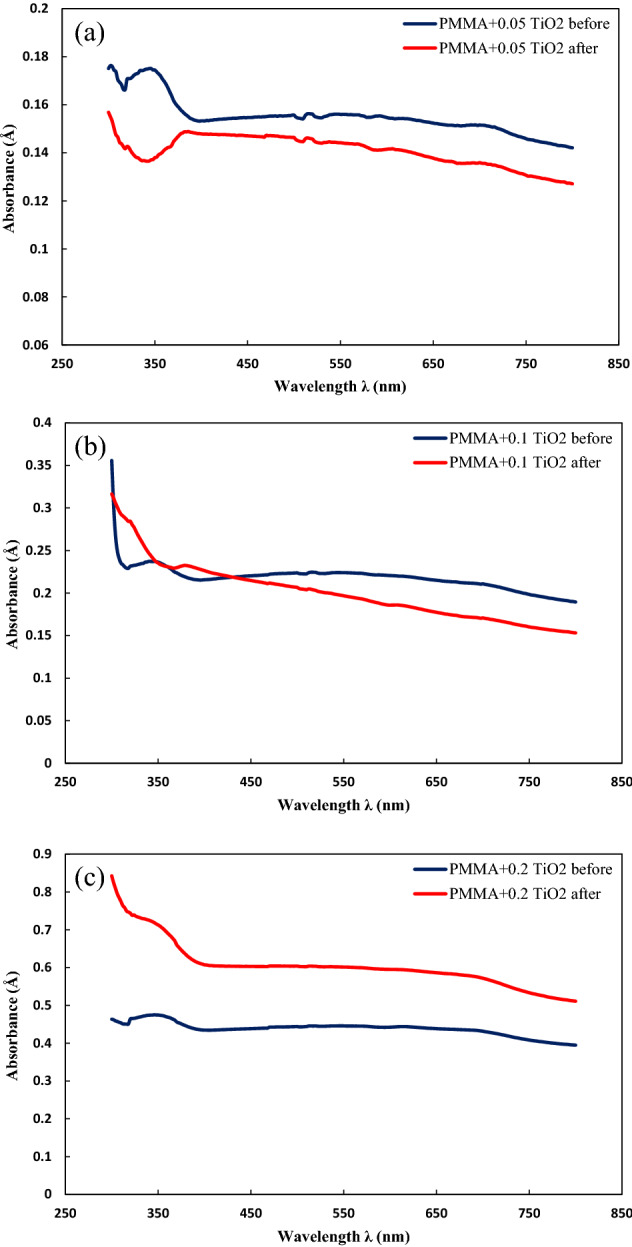
UV–Vis spectra of PMMA coating containing (**a**) 0.05 wt%, (**b**) 0.1 wt%, and (**c**) 0.2 wt% TiO_2_ nanoparticles.

Figure 3 represents the UV–Vis spectra of the prepared PMMA coating containing 0.05 wt%, 0.1 wt%, and 0.2 wt% TiO_2_ nanoparticles before and after being exposed to the UV lamp. Considering the PMMA loaded with 0.05 wt% of TiO_2_ nanoparticles, Fig. 3a, before UV exposure, the absorption at λ = 320 nm was equal to 0.1707 Å, after which the absorption increased. At λ = 344 nm, it reached its highest value, 0.1751 Å. Then the absorption trend was constant up to λ = 346 nm, and after that, it decreased and continued until λ = 398 nm with an absorption of 0.1532 Å. After λ = 398 nm, the absorption amount increased again and peaked at λ = 516 nm with absorbance equal to 0.1563 Å. From this point towards λ = 800 nm, the end of the visible-light region, the obtained value for absorption declined continuously and reached 0.1421 Å. It is also noteworthy that, at the wavelength corresponding to the beginning of visible light’s range (λ = 400 nm), the obtained value of absorption was found to be 0.1533 Å, which is prior to the second peak. Subsequent to the exposure of the polymer film to UV rays and destruction of the polymer film, absorption was measured as 0.1427 Å at λ = 320 nm, the starting point of the ultraviolet A (UVA) range. It was observed that the absorption decreased until λ = 342 nm, after which it increased and reached the peak of 0.1489 Å at λ = 386 nm. Then the absorption pursued a downward trend oscillatorily and was measured as 0.1271 Å at λ = 800 nm. Considering the PMMA with and without coating, it can be comprehended that the loaded nanoparticles increased the absorbance with UV and visible radiation range. This enhancement can be justified by TiO_2_ nanoparticles’ inherent characteristics regarding photons sensing. The electron–hole pair state of the TiO_2_ sites has been proved to be highly reactive; thus, due to the recombination of these pairs, and photogenerated charge carriers, the quantum efficiency of this material is relatively high^32,33^. Xu et al.^21^ investigated the transparency of PC/PMMA coated with TiO_2_ film with granular morphology. It was observed that adding TiO_2_ film could significantly enhance the stability against UV radiation. The remarkable reduction of the UV-exposed sample within λ = 300 nm and λ = 344 nm range can be explained due to the inconsistent dispersion and agglomerated nanoparticles on the surface of PMMA. Agglomeration is one of the major factors regarding reflection capability and transmission; thus, inconsistent dispersion alongside UV irradiation, which enhances the agglomeration process, resulted in reduced absorbance^34,35^.

Figure 3b demonstrates the visible and UV measurement of PMMA coated with 0.1 wt% TiO_2_ nanoparticles prior to and after exposure to UV lamp. The absorption of this material before UV exposure at λ = 300 nm was equal to 0.2316 Å, and it plummeted to 0.2291 Å at λ = 318 nm. After this sharp decrease, the absorption peaked at 0.2373 Å at λ = 340 nm and λ = 346 nm. Consecutively, the obtained values for absorbance decreased and at λ = 400 nm measured as 0.2157 Å. The absorption followed a fluctuating pattern regarding the wavelength range between 400 and 800 nm. The amount of absorbance enhanced until λ = 516 nm and got to 0.2246 Å, after which it was reduced and recorded as 0.1895 Å at λ = 800 nm. Consequent to UV exposure, the highest value for absorption (0.3165 Å) was observed at λ = 300 nm. The absorption decreased at a moderate speed until λ = 320 nm (start of UVA range) and was measured as 0.2845 Å. After this point, the absorbance graph reduced at a higher speed until λ = 366 nm, prior to its second peak at λ = 378 nm and 0.2325 Å. From the second peak onwards, the graph declined almost with a constant slope until λ = 800 nm, at which point the absorbance was recorded as 0.1532 Å. It is also noteworthy that the absorbance value at the start point of the visible light was assessed as 0.2324 Å. Another significant feature is the higher absorbance value after UV exposure between λ = 302 nm and λ = 430 nm, which covers the UVA range and partial range of visible light. This can be justified by the high absorbance of TiO_2_ between 300 and 500 nm under UV light, which Stevanovic et al.^36^ investigated regarding its photoluminescence feature. The second detectable phenomenon is the intensified reduction of absorbance rate for the UV-exposed sample by developing a wavelength of more than 450 nm. TiO_2_ nanoparticles were found to be aggregated during exposure to UV irradiation; moreover, these nanoparticles proved to have low absorbance at higher wavelengths. The reduced light absorption within the visible light range and increasing wavelength can also be explained by the destruction of bonds and enhanced amorphous structure^36–38^.

The UV–Vis spectroscopy for PMMA coated with 0.2 wt% was investigated without and with continuous UV irradiation (Fig. 3c). For the sample without UV exposure, the amount of the absorbed light at λ = 300 nm was 0.4634 Å. The absorbed light slightly increased by increasing wavelength and reaching its best absorbance at 0.4751Å. This feature decreased consecutive to the above-mentioned wavelength and declined to 0.4345Å at λ = 400 nm. The absorbance insignificantly fluctuated within the visible light range but had an overall downward trend reducing to 0.3948 Å at λ = 800 nm. Subsequent to exposure and degradation of polymer composition, the absorbance within the UVA range started at λ = 300 nm with an amount of 0.8427 Å. The absorbed light significantly declined with increasing wavelength until λ = 400 nm, which was 0.6072 Å. The absorbance of this material remained almost steady by the wavelength development until λ = 680 nm and decreased to 0.511 Å at λ = 800 nm. Comparing both graphs, it can be deduced that the higher content of TiO_2_ nanoparticles could not improve the transparency of the prepared coating; furthermore, during exposure to UV irradiation, its UV-shielding characteristics decreased. The higher absorbance can be rationalized due to higher degradation in the structure of the coated polymer film. The possible reason for this phenomenon may be due to nanoparticle agglomeration, which hindered PMMA’s ability to pass light. PMMA is an ultra-white opaque membrane, and it has been proved that the additional loading and large TiO_2_ nanoparticles can reduce the transmitted light in the case of translucent white films. Although TiO_2_ nanoparticles are used to scatter the visible light and impart brightness, whiteness, and opacity, additional nanoparticles would not be preferable in this case based on the aforementioned factors^37,39^.

Considering the acquired UV–Vis spectroscopy of PMMA with different content of TiO_2_ nanoparticles (Table 3), it can be concluded that PMMA including 0.2 wt% TiO_2_ nanoparticles resulted in high absorbance in both visible light and UV light regions, leading to lower transparency of the prepared coating. The major goal of this study was transparency within the visible light range. On this basis, the 0.2 wt% loading was not preferable. Furthermore, after degradation of the sample using lower content of TiO_2_ nanoparticles, 0.05 wt%, the amount of the absorbed light decreased. This can be due to the priorly discussed factors, including the amorphous structure of the polymer film and the non-uniform dispersion of the added nanoparticles in the base polymer film. However, the sample containing 0.1 wt% of TiO_2_ nanoparticles had almost similar performance with or without exposure to UV irradiation, which is a practical and favorable feature. This sample performed higher and lower absorbance within UV and visible light, respectively, which indicated more transparency within the targeted range.

**Table 3:** The obtained absorbance of the prepared coatings using TiO_2_ nanoparticles at different wavelengths.

Coating	Test	Absorbance		
		At λ = 400 nm	At λ = 800 nm	Maximum
PMMA + 0.05 wt% TiO_2_	Before UV exposure	0.1421	0.1533	0.7151
	After UV exposure	0.1271	0.1479	0.1569
PMMA + 0.1 wt% TiO_2_	Before UV exposure	0.1895	0.2157	0.3559
	After UV exposure	0.1532	0.2264	0.3165
PMMA + 0.2 wt% TiO_2_	Before UV exposure	0.3948	0.4345	0.4634
	After UV exposure	0.5110	0.6072	0.8427

#### PMMA + ZnO nanoparticles coating

**Figure 4:**
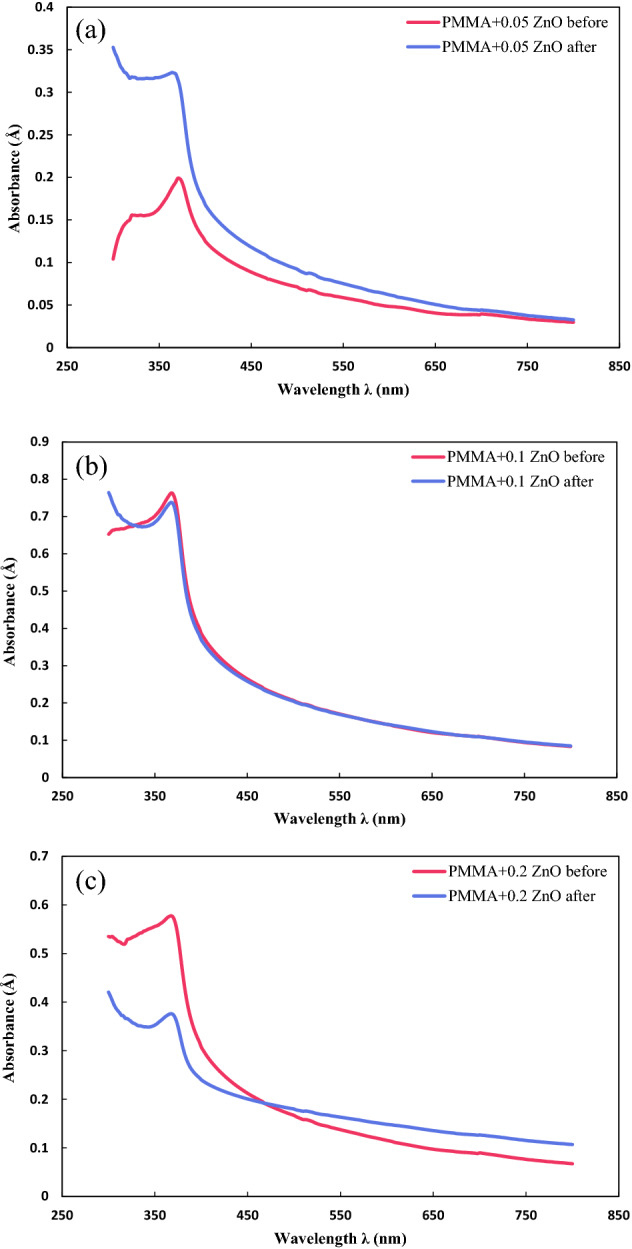
UV–Vis spectra of PMMA coating containing (**a**) 0.05 wt%, (**b**) 0.1 wt%, and (**c**) 0.2 wt% ZnO nanoparticles.

Figure 4 demonstrates the UV–Vis spectra of the prepared PMMA coatings containing 0.05 wt%, 0.1 wt%, and 0.2 wt% ZnO nanoparticles before and after being exposed to the UV lamp. The absorbance of the PMMA with 0.05 wt% coating (Fig. 4a) was 0.1040 Å at λ = 300 nm, after which it reached its peak of 0.1988 Å at λ = 370 nm. Consecutively, the obtained amount continuously decreased and was observed as 0.1251 Å and 0.0298 Å at λ = 400 nm and λ = 800 nm, respectively. One of the significant features of this specimen is that after λ = 430 nm, the absorbance value reached under 0.1 Å. In other words, the absorbance was close to zero and favorable within the visible light range (λ = 400–800 nm). After degradation, the absorbance of the sample was 0.3528 Å at the initial wavelength (λ = 300 nm). The absorption initially decreased, then increased, reaching its peak at λ = 364 nm with a value equal to 0.3232 Å. After this point, the absorbance of the investigated sample sharply declined and reached values of 0.1680Å and 0.0326Å at λ = 400 nm and λ = 800 nm, sequentially. The experiment showed higher absorbance after UV exposure due to the degradation of its structure; however, the absorption difference was almost the same in the visible light of spectra. The performance of the PMMA-based coatings with the addition of ZnO nanoparticles was better than PMMA owing to the fact that ZnO nanoparticles have photoluminescence characteristics due to the excitonic behavior and photo-generated electrons and holes. This characteristic can also be affected by synthesis procedure, surface defects, vacancies, and morphology^40,41^. Soni et al.^42^ confirmed that the addition of ZnO nanoparticles to the polymer films could enhance the optical properties of the prepared coatings.

UV–Vis spectroscopy of the PMMA + 0.1 wt% ZnO nanoparticles is presented in Fig. 4b. The sample without degradation and UV-light exposure had an absorbance of 0.6525 Å at λ = 300 nm. The absorption value then sharply increased and reached the peak of 0.7634 Å at λ = 368 nm. Ensuing this peak point, the absorption continuously decreased until the last examined wavelength. The obtained values for this parameter at the beginning and end of the visible light range (λ = 400 nm and λ = 800 nm) were 0.3876 Å and 0.0834Å, respectively. The degraded sample’s absorbance was 0.7644 Å at λ = 300 nm. It was also noted that within the range of λ = 732–800 nm, the absorbance of this sample was below 0.1 Å. After degradation, the UV absorption was 0.7644 Å at λ = 300 nm, which first decreased to 0.6721 Å by increasing the wavelength to 336 nm within the UVA range. It was then increased and reached its peak at λ = 368 nm with an absorbance of 0.7380 Å, and after this point, it significantly declined to 0.0849 Å at λ = 800 nm. The measured absorbance at visible light’s starting point (λ = 400 nm) was 0.3702 Å, and the absorbance value was lower than 0.1 Å after λ = 734 nm. Although the amount of absorbed light was higher for the PMMA + 0.1 wt% of ZnO nanoparticles compared with the coating containing 0.05 wt% ZnO nanoparticles, the 0.1 wt% PMMA/ZnO nanocoating had a remarkable overlap regarding its absorbance before and after the UV exposure. It was assessed by Senga et al.^37^ that increasing ZnO nanoparticle content could enhance the photoluminescent properties due to the addition of the availability of charge carriers. Moreover, the enhanced absorbance can be attributed to the jump of electrons from the valence band to the empty conduction band^37,42^.

Figure 4c represents the PMMA coating with the addition of 0.2 wt% of ZnO nanoparticles. It can be seen that absorption initially increased from 0.5351 Å at λ = 300 nm to 0.5774 Å at λ = 368 nm, which is the maximum point of the obtained graph. From the peak point onward, the absorbance dramatically reduced to 0.3074 Å at λ = 400 nm and 0.0673 Å at λ = 800 nm. The absorption of the provided sample was near zero (< 0.1 Å) at wavelengths higher than 642 nm. The degraded sample with UV lamp peaked at 0.3759 Å at λ = 368 nm after its decrease from 0.4206 Å at λ = 300 nm. By additional wavelength development, it was observed that the absorption amount decreased at a slower rate compared to the not-degraded sample. It was reduced to 0.2401 Å and 0.1068 Å at 400 nm and 800 nm wavelengths, respectively. According to both graphs, it can be concluded that the absorption of the sample degraded with the UV lamp was relatively lower than the sample without UV exposure within the UVA region; however, its absorbance was higher within the visible light spectra. It may be due to the severe destruction of the sample’s structure, which causes severe destruction of its bonds and hinders its photoluminescence feature in contact with visible light. Therefore, despite its high transparency, the UV lamp severely destroys the ZnO nanoparticle and loses its original structure and transparency^43^.

Table 4 lists the obtained absorbance of the prepared coatings using ZnO nanoparticles at different wavelengths. Considering PMMA/ZnO nanocoatings, it was seen that the absorption of 0.2 wt% PMMA/ZnO nanocoating was lower than 0.1 wt% one within the UVA region; however, the absorption was higher within the visible light range. Thus, the coating containing 0.1 wt% ZnO nanoparticles showed higher transparency with visible light spectra, and 0.2 wt% coating was inappropriate. Although the lower content of ZnO nanoparticles (0.05 wt%) demonstrated favorable photoluminescence features within the visible light range, it had low absorption within the UV-light range, which could not fulfill the goal of this study. The 0.1 wt% ZnO nanocoating showed high adsorption in the UV range before and after degradation. Furthermore, this sample showed absorbance lower than 0.1 Å at visible light spectra.

**Table 4:** The obtained absorbance of the prepared coatings using ZnO nanoparticles at different wavelengths.

Coating	Test	Absorbance		
		At λ = 400 nm	At λ = 800 nm	Maximum
PMMA + 0.05 wt% ZnO	Before UV exposure	0.1251	0.0298	0.1978
	After UV exposure	0.1680	0.0326	0.3528
PMMA + 0.1 wt% ZnO	Before UV exposure	0.3876	0.0834	0.7634
	After UV exposure	0.3702	0.0849	0.7644
PMMA + 0.2 wt% ZnO	Before UV exposure	0.3074	0.0673	0.5774
	After UV exposure	0.2401	0.1068	0.4206

#### PMMA + nanohybrid coating

**Figure 5:**
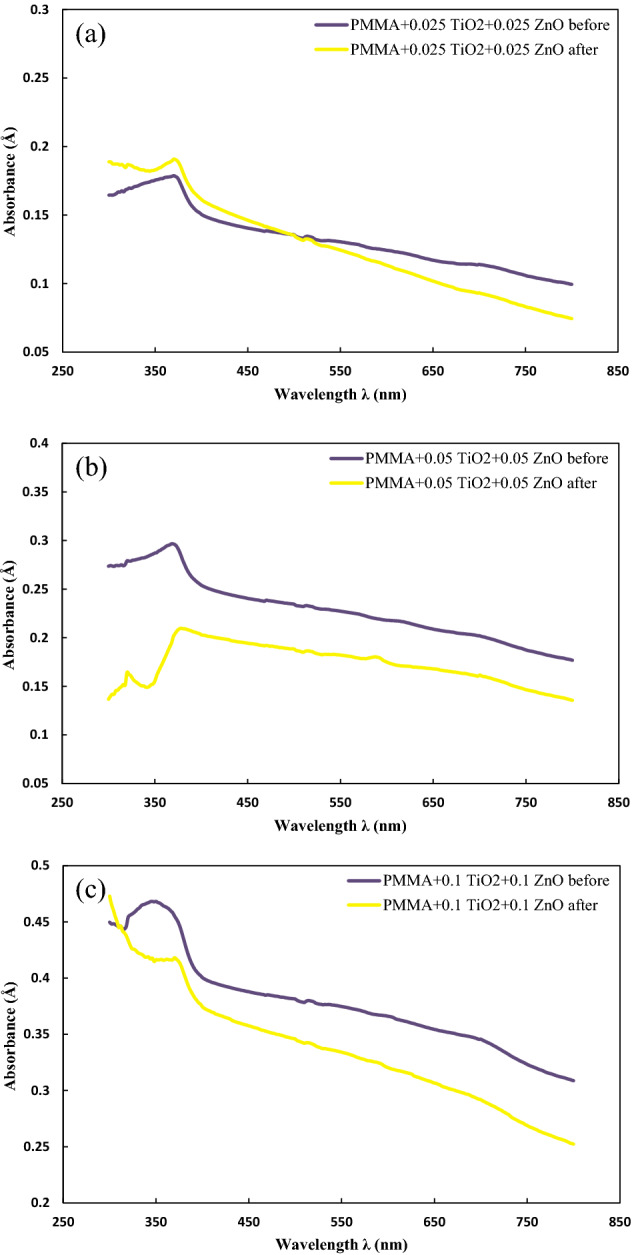
UV–Vis spectra of PMMA coating containing (**a**) 0.025:0.025 wt%, (**b**) 0.05:0.05 wt%, and (**c**) 0.1:0.1 wt% of TiO_2_:ZnO nanoparticles.

In order to investigate the hybrid coatings, TiO_2_:ZnO nanoparticles were added with different wt% ratios, including 25:75, 30:70, 50:50, 70:30, and 75:25. After conducting the experiments considering different ratios, the optimal ratio was 50:50 wt% of TiO_2_:ZnO; thus, this ratio was chosen for the rest of the study. TiO_2_/ZnO nanohybrids were added to PMMA at three different contents, 0.025 wt%, 0.05 wt%, and 0.1 wt%, and the UV–Vis spectroscopy with and without UV-lamp was conducted to measure their absorbance characteristics. The UV–vis spectra of the prepared nanohybrid PMMA-based coatings utilizing TiO_2_ and ZnO nanoparticles at diverse contents are exhibited in Fig. 5. Figure 5a shows the UV–vis spectra of nanohybrid coating at 0.025 wt% content of each nano-additives before and after being exposed to the UV light. The sample’s absorbance at λ = 300 nm before and after degradation were 0.1645 Å and 0.1888 Å, respectively. The absorbance trend of both prepared PMMA-based nanohybrids increased and peaked at λ = 370 nm and reached 0.1787 Å and 0.1908 Å for the sample without and with degradation, sequentially. Subsequent to the peak, both graphs showed a decreasing trend with the increase of the wavelengths; however, degradation accelerated the decline of absorbance. The sample without degradation was an absorbance value of 0.1504 Å at λ = 400 nm and 0.0994 Å at λ = 800 nm. With exposure to the UV lamp, the absorption of the sample was 0.1613 Å and 0.0744 Å at λ = 400 nm and λ = 800 nm. The significant feature that can be apprehended was that the absorbance below 0.1 Å was observed after λ = 658 nm for degraded and λ = 798 nm for non-degraded samples. The behavior regarding absorbance within the UVA region can be attributed to the presence of ZnO nanoparticles like it was investigated regarding 0.05 wt% of ZnO nanoparticles and the availability of charge carriers. Moreover, the nanohybrid coatings had relatively low absorbance due to the presence of TiO_2_ nanoparticles and their photoluminescence and reflecting properties.

The UV–Vis spectroscopy of the PMMA nanohybrid coatings utilizing 0.05 wt% of the considered nano-additives is presented in Fig. 5b. Prior to UV exposure, the sample had absorption of 0.2735 Å at λ = 300 nm and increased to 0.2966 Å at λ = 3368 nm. By increasing the wavelength to 400 nm, absorbance sharply declined to 0.2540 Å at λ = 400 nm, then it was slowly reduced towards 0.1768 Å at λ = 800 nm. After degradation, the absorbance was raised from 0.1367 Å at λ = 300 nm to its first peak with an absorbance of 0.1645 Å at λ = 320 nm. From that point onward, the absorption peaked at λ = 378 nm with absorbance of 0.2097 Å. Subsequently, the absorption decreased with a moderate speed to 0.2030 Å and 0.1356 Å at λ = 400 nm and λ = 800 nm, respectively. Overall, it can be seen that both samples had no absorbance below 0.1 Å due to the presence of TiO_2_ nanoparticles. The 0.2 wt% nanohybrid coating (0.1 wt% of each TiO_2_ and ZnO nanoparticles) was tested using UV–Vis, and the obtained results are presented in Fig. 5c. Before degradation, the absorbance was 0.4497 Å at λ = 300 nm and increased to 0.4682 Å at λ = 346 nm. With rising the wavelength, the absorbance decreased to 0.4004 Å at λ = 400 nm and then to 0.3087 Å at λ = 800 nm. The 0.2 wt% nanohybrid coating degraded with the UV lamp had an absorbance of 0.4729 Å at λ = 300 nm, which sharply decreased by increasing wavelength to 0.3744 Å at λ = 400 nm. Subsequently, the sample's absorption decreased and reached 0.2535 Å at λ = 800 nm. It is also noteworthy that this sample did not have absorbance below 0.1 Å; thus, this coated polymer film could not show acceptable transparency.

Table 5 lists the obtained absorbance of the prepared coatings using hybrid nanoparticles at different wavelengths. By reducing the content of nanoparticles in the hybrid coated PMMA, absorption in the UVA region decreased; thus, more polymer film with higher wt% was favorable. However, by comparing these samples with pure TiO_2_ or ZnO coating, it could be observed that the absorption amount was less, which is not favorable for the intended goals of this study. Considering the transparency within the visible light region, samples with 0.05 wt% and 0.1 wt% loading TiO_2_ and ZnO had absorbances above 0.1 Å. These results proved that they did not have targeted transparency. The only hybrid sample with acceptable absorbance in the visible light spectra was attributed to 0.05 wt% equal loading of each nanoparticle. However, it had lower absorbance in the UVA region. However, comparing the best results aligned with the purpose of the study, attributed to 0.1 wt% of ZnO, much less amount of ZnO and TiO_2_ was required to obtain nearly the same results. The above-mentioned factor is of importance when mass production is considered. The lower light absorption in the UVA range could be attributed to TiO_2_ nanoparticles. High absorbance in visible light can be explained by the acceleration of structural destruction due to the reflecting properties of TiO_2_. Overall, it could be deduced that the top two coatings for PMMA were 0.1 wt% of ZnO and 0.025:0.025 wt% of TiO_2_: ZnO.

**Table 5:** The obtained absorbance of the prepared coatings using TiO_2_/ZnO nanohybrid at different wavelengths.

Nanohybrid coating	Test	Absorbance		
		At λ = 400 nm	At λ = 800 nm	Maximum
PMMA + TiO_2_/ZnO (0.025/0.025) wt%	Before UV exposure	0.1504	0.0994	0.1787
	After UV exposure	0.1613	0.0744	0.1908
PMMA + TiO_2_/ZnO (0.05/0.05) wt%	Before UV exposure	0.2540	0.1768	0.2966
	After UV exposure	0.2030	0.1356	0.2097
PMMA + TiO_2_/ZnO (0.1/0.1) wt%	Before UV exposure	0.4004	0.3087	0.4682
	After UV exposure	0.3744	0.2535	0.4729

### FT-IR analyses

**Figure 6:**
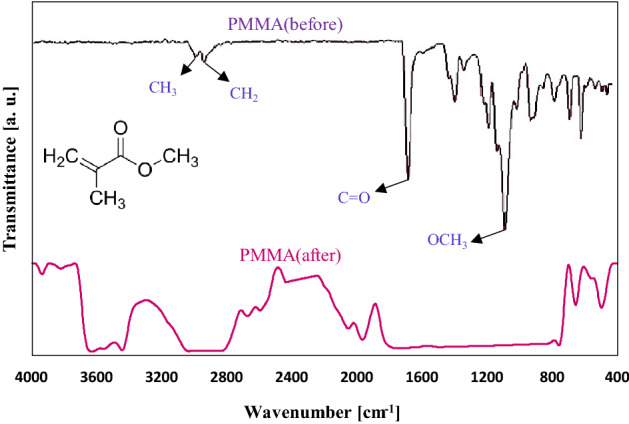
FT-IR spectra of pure PMMA coating before and after degradation.

The complexation or coordination of the dopant nanoparticles with the host polymer was studied utilizing the FTIR spectroscopy technique^44,45^. Figure 6 shows the FT-IR spectra of PMMA film before and after degradation. As can be seen before degradation, the stretching vibration of OCH_3_ is observed at 1190 cm^−1^. The peak seen at 1019 cm^−1^ was attributed to the ether lone pair^46^. The asymmetric stretching of C-H existing in the structure of CH_3_ and CH_2_ were observed at 2990 cm^−1^ and 2900 cm^−1^, respectively. The intense sharp peak of carbonyl stretching of the pendent group (COOCH_3_) was beholded at about 1720 cm^−1^. Besides, the peaks at 1436 cm^−1^, 1400 cm^−1^, and 1380 cm^−1^ were allocated to the deformation modes of CH_3_. Two obviously seen bands at 1150 cm^−1^ were assigned to the CH_3_ twisting. It is obvious that the bands at 995 cm^−1^ and 983 cm^−1^ corresponding to the C–C stretching vibration modes. Meanwhile, the bands realized at 1238 cm^−1^, 1190 cm^−1^, and 1150 cm^−1^ were associated with the C-O stretching vibration (ester linkage). A methylene rocking peak was observed at approximately 769 cm^−1^^47^.

Considering the FT-IR spectrum of the pure polymer film after degradation, the peaks corresponding to the region of 3600–3400 cm^−1^ were related to the hydroxyl functional group (stretching R–OH). Here, hydroxyl functional groups were formed due to the compound’s strong tendency to bond with water vapor molecules in the environment^48^. A new peak with low intensity at 2750 cm^−1^ was ascribed to the symmetric stretching of C–H in CH_3_^47^. Two peaks related to CH_3_ and CH_2_ broadened from 3100 cm^−1^ to 2800 cm^−1^, which could confirm the degradation of PMMA coating film after being exposed to the UV lamp. Moreover, symmetric stretching of C-H existing in the CH_3_ group was not changed after degradation, which can be seen at 2750 cm^−1^. It is worth noting that the CH_3_ deformation, CH_3_ twisting, C–C, and C-O stretching vibration underwent degradation due to the UV rays and vapor droplets. This might have affected the functional groups of the film and caused a broad band ranging from 1850 cm^−1^ to 850 cm^−1^. In addition, the methylene rock peak was shifted from 769 cm^−1^ to 800 cm^−1^.

Generally, considering the FT-IR of PMMA film coating, with decreasing in the intensity of degraded polymer film, peak shifting, and broadening the bands, it was confirmed that the pure polymer degraded after 720 h and should fortify the ability of film to resist when exposing to the UV irradiation with adding of proper nanoparticles.

**Figure 7:**
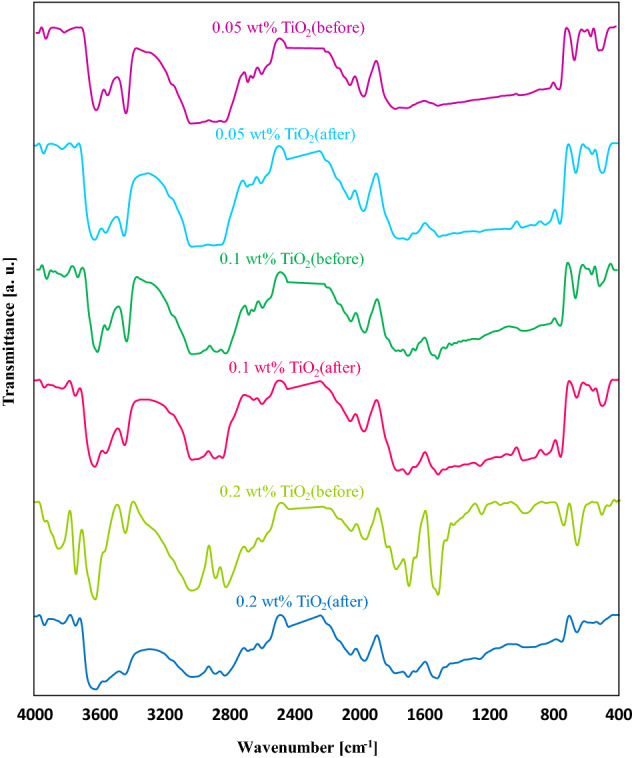
FT-IR spectra of PMMA/TiO_2_ nanocoating before and after degradation.

In the following, the samples containing TiO_2_, ZnO, and TiO_2_/ZnO nanoparticles (hybrid) as additives at different concentrations were examined before and after exposure to ultraviolet lamp. The FT-IR spectra of the PMMA coating film containing 0.05 wt%, 0.1 wt%, and 0.2 wt% TiO_2_ nanoparticles before and after being exposed to the UV lamp is demonstrated in Fig. 7. Before degradation of PMMA/TiO_2_ nanocoating films, the wide peak seen at approximately 1650 cm^−1^ was attributed to the Ti–OH due to the absorption of the hydrogen group of water on the surface of the TiO_2_ nanoparticles. Asymmetric and symmetric stretching vibrations of a hydroxyl group may range from 3600 cm^−1^ to 3400 cm^−1^^49^. It is worth noting that the intensity of hydroxyl stretching was not altered for 0.05 wt% and 0.1 wt%, while the intensity of 0.2 wt% TiO_2_ nanoparticles peaks was changed and strengthened due to their tendency to adsorption of the water molecule. Meanwhile, the Ti–OH band could not be observed for 0.05 wt% TiO_2_ nanoparticles because of the lower amount of used TiO_2_ nanoparticles and weak interaction between TiO_2_ nanoparticles and PMMA. However, this band was obviously seen for 0.1 wt% and 0.2 wt% TiO_2_ nanoparticles because of the excessive amount of utilized TiO_2_ nanoparticles in the reaction environment. The OH stretching vibration revealed several sharp peaks, denoting the presence of different types of isolated Ti–OH groups on the surface. In addition, the intense bands seen at 756 cm^−1^, 657 cm^−1^, and 489 cm^−1^ ascribed to the Ti–O stretching vibration and confirmed the existence of TiO_2_ nanoparticles. The intensity of these peaks diminished compared to the absolute polymer matrix, which indicated the remarkable miscibility of nanoparticles and polymer. Considering the effect of nano additives concentration, it can be said that with increasing the content of TiO_2_ nanoparticles, the intensity of Ti–O–Ti stretching bands was reinforced, which can be observed at 1483 cm^−1^^46,50^. The peak realized at 1510 cm^−1^ represents the antisymmetric vibration of the (COO^-^) group that exists in the structure of the ester group^51^. This band was sharper for the samples containing 0.1 and 0.2 wt% TiO_2_ nanoparticles. However, the intensity of the COO^-^ group for the PMMA/0.1 wt% TiO_2_ nanoparticles sample content was low.

Considering the acquired FT-IR spectra of PMMA with different content of TiO_2_ nanoparticles, it is significant that the intensity of coating film containing 0.05 wt% TiO_2_ was low compared to the others; hence, the peaks remained consistent after degradation. In contrast, the film containing 0.1 wt% had sharp peaks and indicated that the PMMA polymer could interact with TiO_2_ comprehensively. The mentioned film stayed unchanged after 720 h exposure to the UV rays, which can be considered as a good choice for UV-shielding application. The intensity of PMMA/0.2 wt% TiO_2_ nanoparticles specimen was higher than others due to the consumed TiO_2_ nanoparticles. Interestingly, the degradation of this film was higher than others leading to the destruction of functional groups, and finally, broadening happened that demonstrated the degradation phenomena. Overall, it can be concluded that PMMA including 0.2 wt% TiO_2_ nanoparticles was not preferable due to the lower resistance when exposed to the UV lamp. On the other hand, PMMA including 0.1 wt% TiO_2_ nanoparticles was not degraded even after 720 h, which exhibited better performance. Meanwhile, owing to the lower content of used TiO_2_ nanoparticles in PMMA containing 0.05 wt% TiO_2_, this sample had almost similar performance with or without exposure to UV irradiation. Therefore, it was not considered a good choice for the considered application.

**Figure 8:**
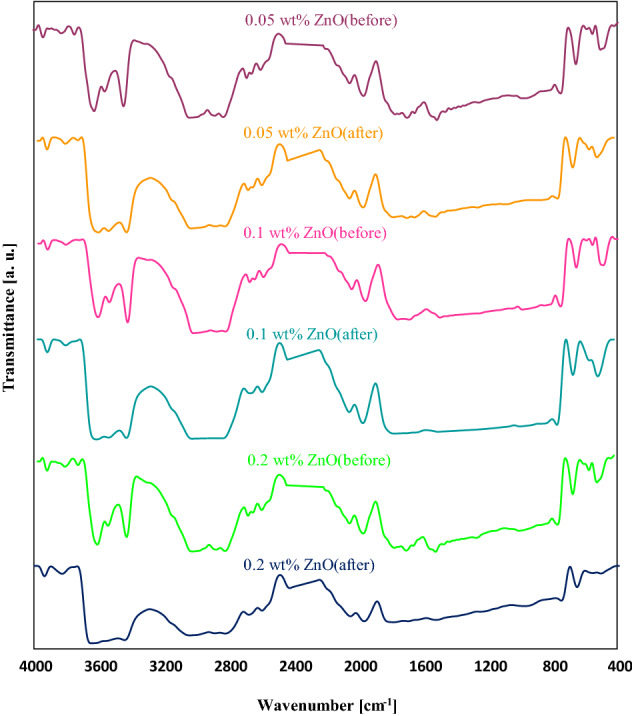
FT-IR spectra of PMMA/ZnO nanocoating before and after degradation.

Figure 8 illustrates the FTIR spectrum of PMMA coating using 0.05 wt%, 0.1 wt%, and 0.2 wt% ZnO nanoparticles before and after being exposed to the UV lamp. Metal oxides usually develop absorption bands in the fingerprint region below 1000 cm^−1^, which are caused by interatomic vibrations. It can be inferred from the FT-IR outcomes of the samples before UV irradiation that the peaks detected at 3600 cm^−1^–3400 cm^−1^ for PMMA/ZnO nanoparticles samples might be the O–H stretching that corresponded to the H_2_O molecule adsorption on the ZnO surface. The OH stretching vibration represents diverse, intense peaks, signifying the attendance of various types of Zn–OH groups on the surface. The bands observed at 507 cm^−1^ and 1690 cm^−1^ were allocated to the distortion and stretching vibration of ZnO nanoparticles, sequentially^52^. The seen band at 1506 cm^−1^ was ascribed to the metal-OH bending vibration (Zn–OH)^48,53^. Surprisingly, the intensity of the peaks and their position enhanced with the increase of ZnO concentration from 0.05 to 2 wt%. There was a slight shift appeared in its position from 769 cm^−1^ to 754 cm^−1^ allocated to the rocking of CH_2_ vibration. Moreover, the peak intensity of PMMA/ZnO was decreased owing to the overlapping between the PMMA and ZnO nanoparticles’ functional bands. It becomes clear that by adding ZnO nanoparticles to the PMMA film, the position and intensity of peaks were changed, demonstrating the good interaction between the pure polymer and ZnO nano additive.

The FTIR of PMMA + 0.05 wt% ZnO nanoparticles after degradation shows that the intensity of ZnO peaks decreased, and OH stretching vibration was destroyed. A broadening phenomenon occurred between 3028 cm^−1^–2830 cm^−1^ and 1778 cm^−1^–800 cm^−1^. It is clear that for PMMA + 0.1 wt% ZnO nanoparticles after degradation, the peak intensity of all functional groups was raised with adding ZnO nanoparticles, while destruction of main bands happened at 3025 cm^−1^–2820 cm^−1^ and 1797 cm^−1^–780 cm^−1^. Considering PMMA/0.2 wt% ZnO nano-coatings, it was seen that a higher amount of utilized ZnO could affect the resistance ability of polymer film. After exposure to the UV rays, all of the functional adsorption groups of this film were devastated, and the band intensity was reduced more than the other. Considering the PMMA containing 0.2 wt% ZnO nanoparticles, the peak intensity and broadening of peaks occurred more than PMMA containing 0.05 wt% and 0.1 wt% ZnO nanoparticles. Furthermore, comparing the PMMA containing 0.05 wt% and 0.1 wt% ZnO nanoparticles, the lower broadening phenomena happened, and drastic changes were seen. Since the optimum sample could be considered definitively from these bands; based on the reasons mentioned in the UV section, PMMA containing 0.1 wt% ZnO nanoparticles was selected as the best sample. On this basis, PMMA + 0.1 wt% ZnO nanoparticles suggested using as UV-protector or shield.

**Figure 9:**
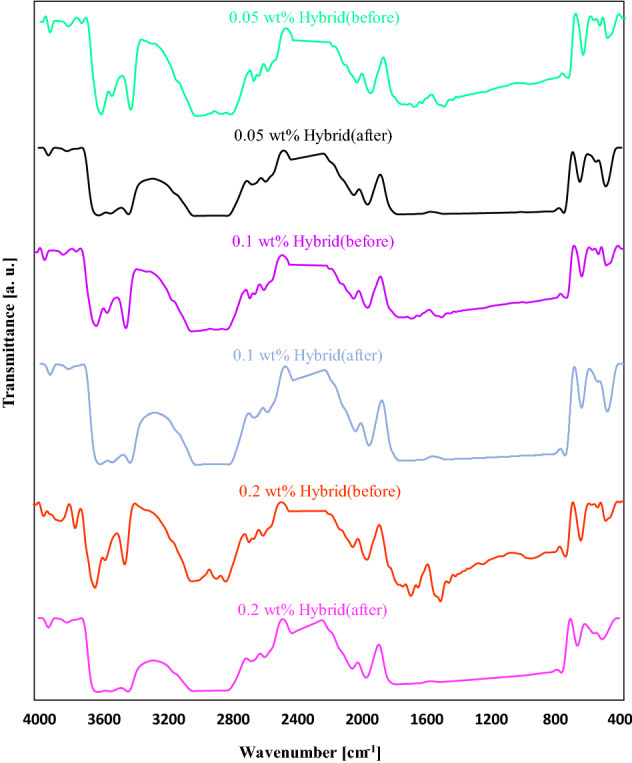
FT-IR spectra of PMMA/nanohybrid (TiO_2_:ZnO) coating before and after degradation.

To examine the effect of hybrid nano additives on the resistance of polymer-based films exposed to the UV lamp, TiO_2_/ZnO nanoparticles were added to the pure PMMA. Figure 9 depicts the FTIR spectra of PMMA containing TiO_2_/ZnO nanoparticles at diverse concentrations. As mentioned in the UV analyses, the optimal ratio was 50:50 wt% of TiO_2_/ZnO; thus, this ratio was chosen for the rest of the study. TiO_2_/ZnO nanohybrids were added to PMMA polymer film at three different contents, including 0.05 wt% (0.025:0.025 wt% of TiO_2_: ZnO), 0.1 wt% (0.5:0.5 wt% of TiO_2_:ZnO), and 0.2 wt% (0.1:0.1 wt% of TiO_2_:ZnO). Fourier transform infrared spectroscopy was employed with and without UV-lamp to investigate their functional groups’ characteristics.

To investigate the durability and chemical stability of the prepared hybrid films, we irradiated the PMMA/ZnO nanocoating in a controlled UV environment for 720 h. As can be deduced from the graphs, overlapping between ZnO and TiO_2_ nanoparticles happened that causing the intensity changes. Before exposure to the UV lamp, the intensity bands lower than 1000 cm^−1^, attributed to the metal oxides, were diminished. In contrast, after degradation, the peak intensity escalated and shifted to higher wavenumbers. With the addition of nano additives into the PMMA matrix, the peaks became sharp and more intense than other hybrids. Considering the PMMA + 0.2 wt% nanohybrid coating, the adsorption group intensity decreased, and the broadening occurred more than in other hybrids. Thus, this coated polymer film could not show good resistance. Comparing two hybrids with each other, it can be said that both hybrids (0.05 wt% and 0.1 wt%) demonstrated almost the same results and a little change in functional groups. However, as mentioned in the UV section, comparing the best results aligned with the aim of the study, a much lower amount of ZnO and TiO_2_ (0.025:0.025 wt% of TiO_2_:ZnO) was required to obtain nearly identical results. The above factor is of importance when mass production is considered. Notably, hybrids film indicated a favorable result when irradiated by the UV lamp.

In total, different nanocoating films were prepared in this study to examine their UV-shielding and resistance capabilities while exposed to UV irradiation. The outcomes revealed that the pure polymer matrix could not show resistance, and all functional groups were degraded after exposure to the UV rays. Among diverse prepared samples, PMMA + 0.1 wt% TiO_2_ nanoparticles, PMMA + 0.1 wt% ZnO nanoparticles, and PMMA + 0.05 wt% nanohybrid coatings were reported as the optimal specimens owing to their great performance to show UV-shielding property after being exposed to the UV lamp. The sample containing 0.05 wt% nanohybrid additives could be a good choice for this study since it exhibited a lower degradation rate compared to other examined samples.

### XRD measurements

**Figure 10:**
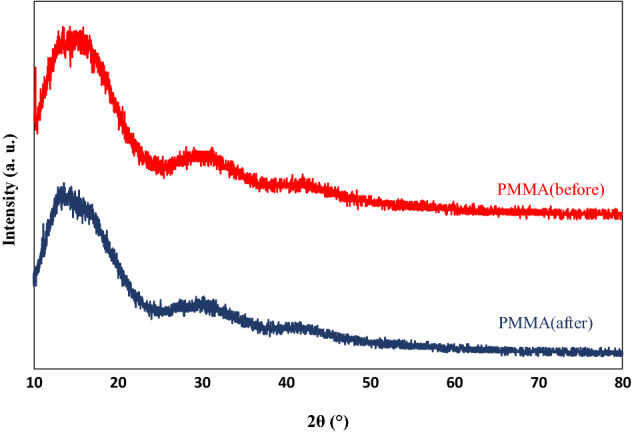
XRD patterns of PMMA coating before and after degradation.

**Figure 11:**
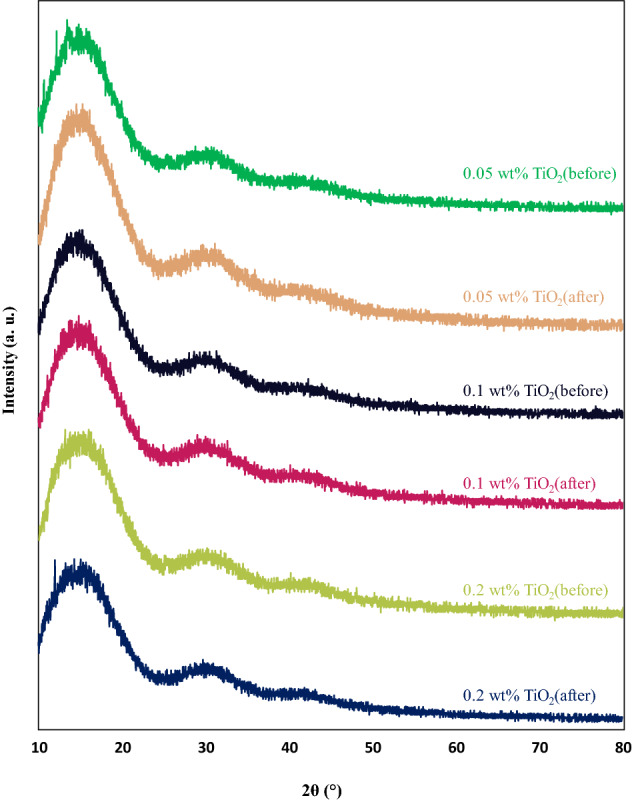
XRD patterns of PMMA/TiO_2_ nanocoatings at different contents before and after degradation.

**Figure 12:**
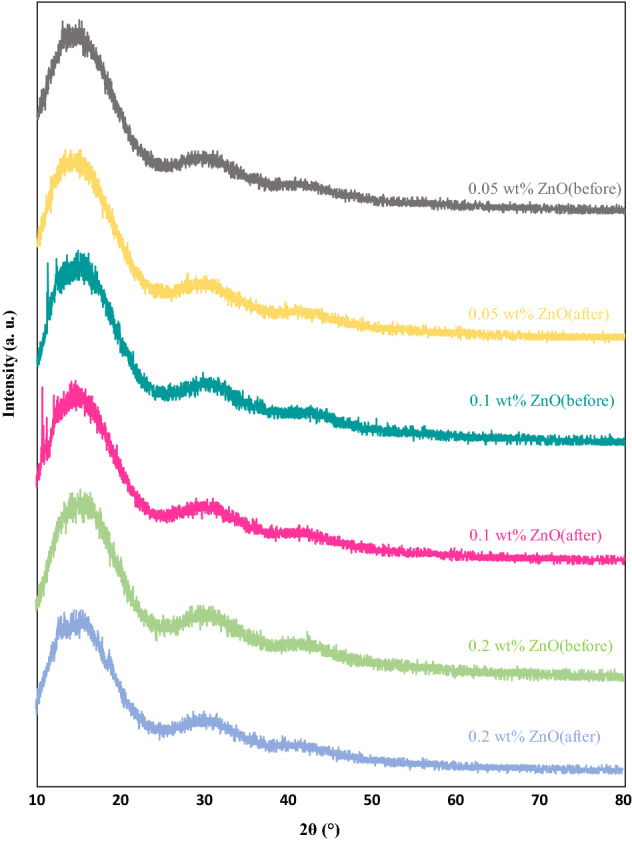
XRD patterns of PMMA/ZnO nanocoatings at different contents before and after degradation.

XRD is one of the most important characterization tools utilized in materials science to easily determine the size and shape of the unit cell for any compound^54^. The XRD patterns were seen without any sharp diffraction peaks, confirming the amorphous nature of the polymer, owing to the attendance of the bulky pendant ester group in the chain of PMMA^55^, as illustrated in Figs. 10, 11, 12. Figure 10 shows the XRD patterns of pure PMMA polymer coating film. It is notable that the intense peak in the XRD patterns of PMMA before degradation was observed at 2θ = 15° due to the amorphous nature of the PMMA matrix, and this peak was shifted to 2θ = 14.01° after exposure to the UV rays^46^. A larger halo at about 15° attributed to the packing of the expanded polymer chain, and a lower wide, intense halo at about 29.69° denoted the order within the main chain^37^. Characteristic diffraction peaks at 2θ values of 14.01°, 29.69°, and 41.93° allocated to the (111), (112), and (211) planes, sequentially. Moreover, the wide humps observed in the XRD spectrum demonstrated the presence of crystals with very low dimensions^54^. It is obvious that the diffraction peak observed at 2θ = 29.69° was shifted to the 2θ = 30.87°, and the peak became wider after exposure to the UV lamp.

Figure 11 represents the XRD diffractions of PMMA loading with 0.05 wt%, 0.1 wt%, and 0.2 wt% TiO_2_ nanoparticles. Considering nanocoating’s XRD patterns, which proves their amorphous nature of them, it is clear that TiO_2_ nanoparticles as an additive could not have the chance to enhance the structural properties of PMMA coating film, which is owing to the fact that TiO_2_ nanoparticles were physically mixed and apparently no chemical reaction took place between the two components. It is worth noting that all of the diffraction patterns were similar to those reported in the literature for PMMA^56^.

Figure 12 demonstrates the XRD diffractions of PMMA film, including 0.05 wt%, 0.1 wt%, and 0.2 wt% ZnO nanoparticles additive. No peaks were referring to the crystalline phase of ZnO nanoparticles in the XRD patterns of the PMMA + ZnO nanocoating films investigated here, which means that during the preparation process, the ZnO nanoparticles were comprehensively dispersed in the solutions. Vigorous bounds exist between the C=O of the PMMA matrix and Zn^2+^, inducing the complete separation of ZnO. The addition of ZnO nanofiller to the PMMA polymer host changed the width and intensity of the primary peaks. This increase affected the crystallinity of the polymer in width or diminished the intensity due to the polymer nanofiller^57,58^.

**Figure 13:**
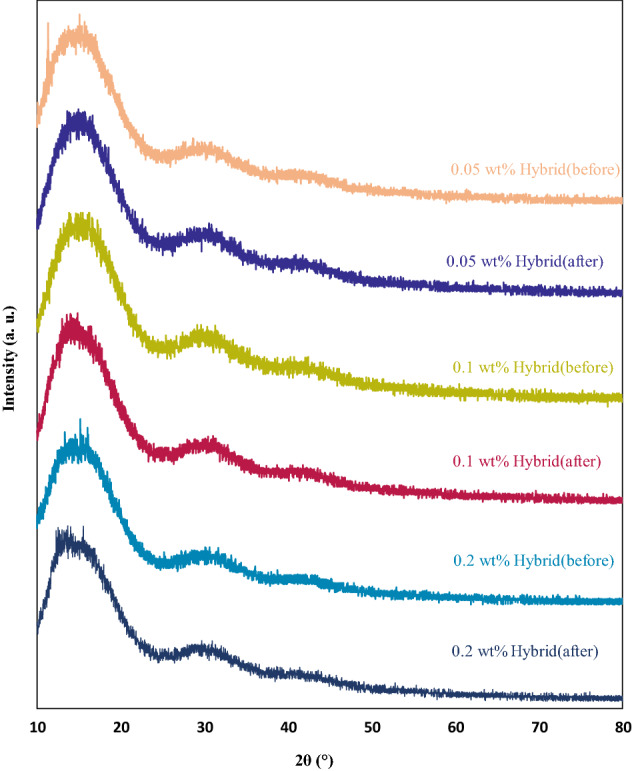
XRD patterns of nanohybrid coatings at different contents before and after degradation.

As stated before, 50:50 wt% of TiO_2_: ZnO was selected as the optimal ratio for the rest of the study. Figure 13 exhibits the PMMA containing (0.025:0.025 wt% of TiO_2_: ZnO), 0.1 wt% (0.5:0.5 wt% of TiO_2_: ZnO), and 0.2 wt% (0.1:0.1 wt% of TiO_2_: ZnO) nanohybrids. As mentioned, no peaks referred to the crystalline phase of ZnO or TiO_2_ nanoparticles in the XRD patterns of the PMMA + hybrid nanocoating films studied here. All of the patterns observed were related to the PMMA coating film.

### SEM, EDX, and histogram

**Figure 14:**
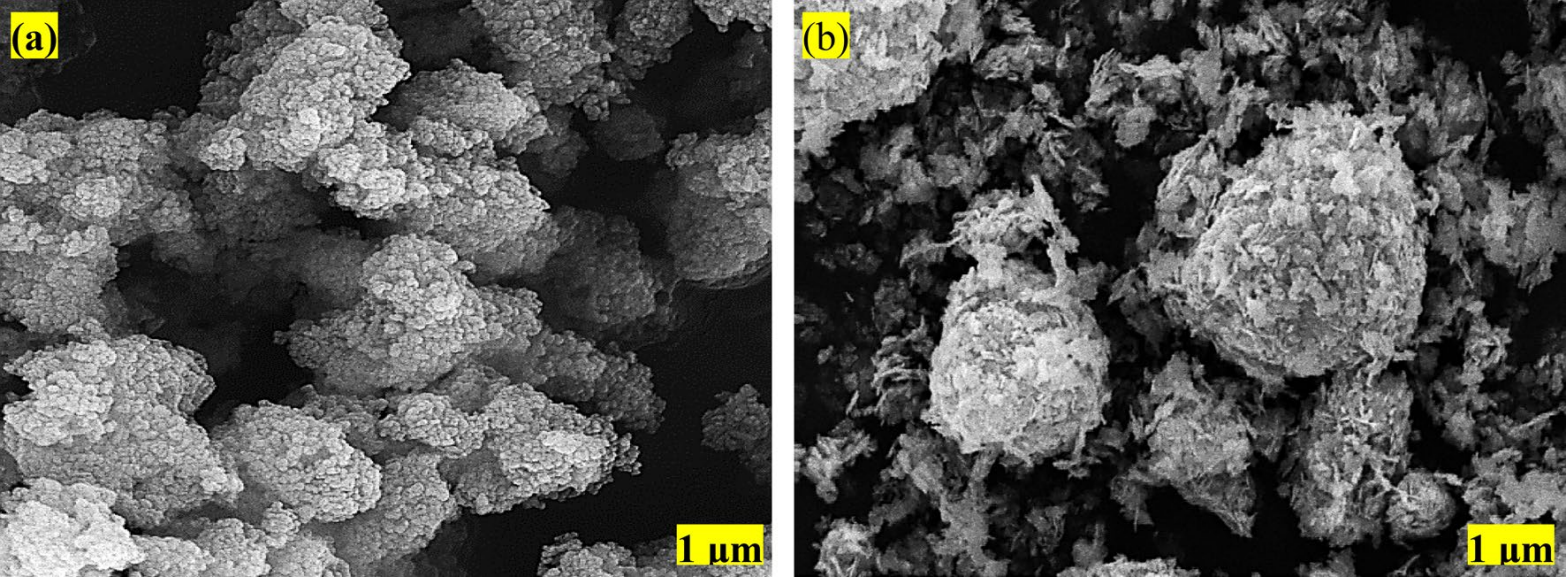
SEM images of (**a**) TiO_2_ and (**b**) ZnO nanoparticles.

Figure 14 represents the scanning electron microscope (SEM) pictures of the used TiO_2_ and ZnO nanoparticles samples. Figure 14a clearly illustrates the well-dispersed spherical-shaped anatase TiO_2_ nanoparticles. As can be seen from SEM images of the TiO_2_ nanoparticles, nanoparticles showed highly heterogeneous structures. These nanoparticles demonstrated denser particles and were prone to be agglomerated. Figure 14b shows the SEM micrographs of ZnO nanoparticles. It is obvious that the nanoparticles were spherical shape nanoparticles.

**Figure 15:**
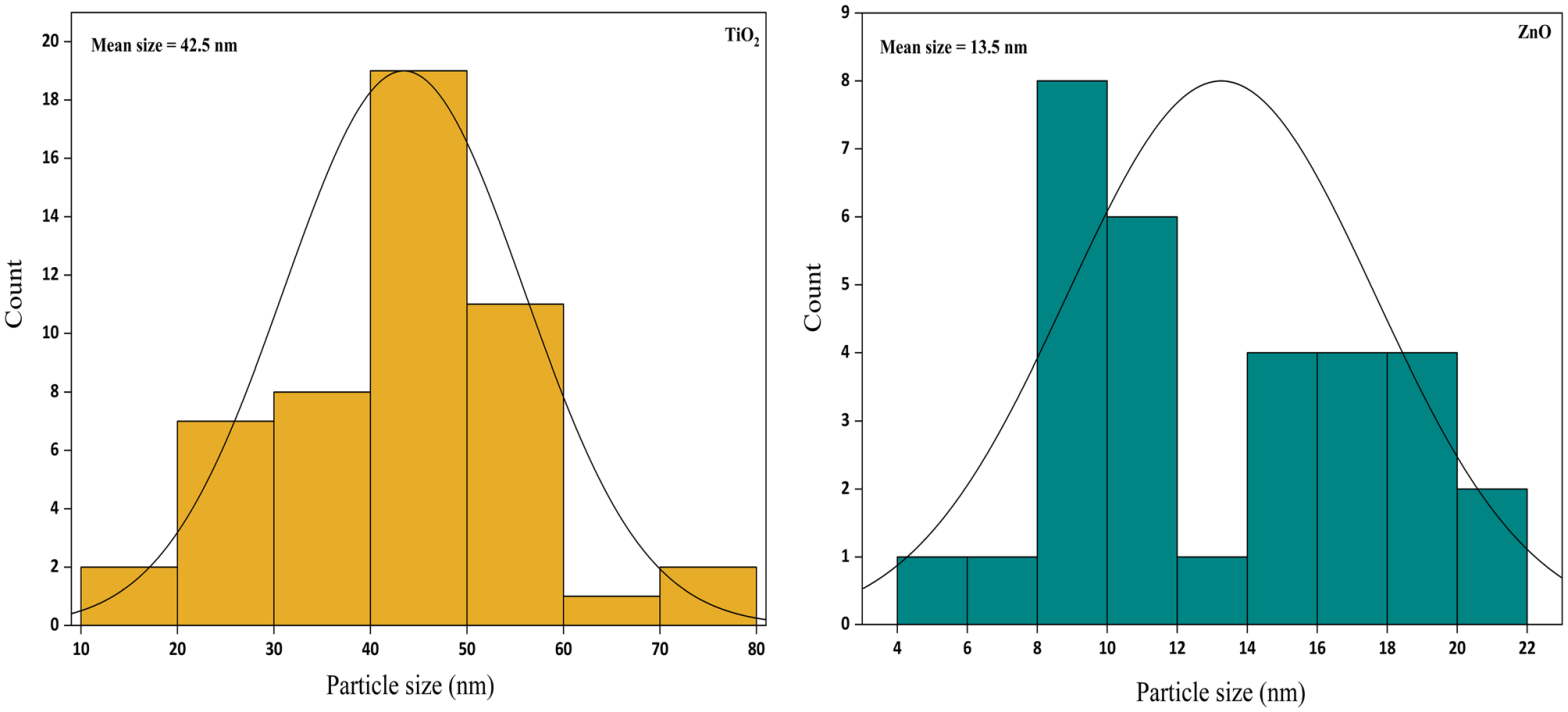
Size distribution of TiO_2_ and ZnO nanoparticles.

Figure 15 illustrates the histograms for TiO_2_ and ZnO nanoparticles. The mean nanoparticle size of TiO_2_ and ZnO nanoparticles was calculated using ImageJ software by measuring at least 40 individual particles. The average diameter size was 42.5 nm for TiO_2_ nanoparticles and 13.5 nm for ZnO nanoparticles. These diameter sizes are in good agreement with Table 1.

**Figure 16:**
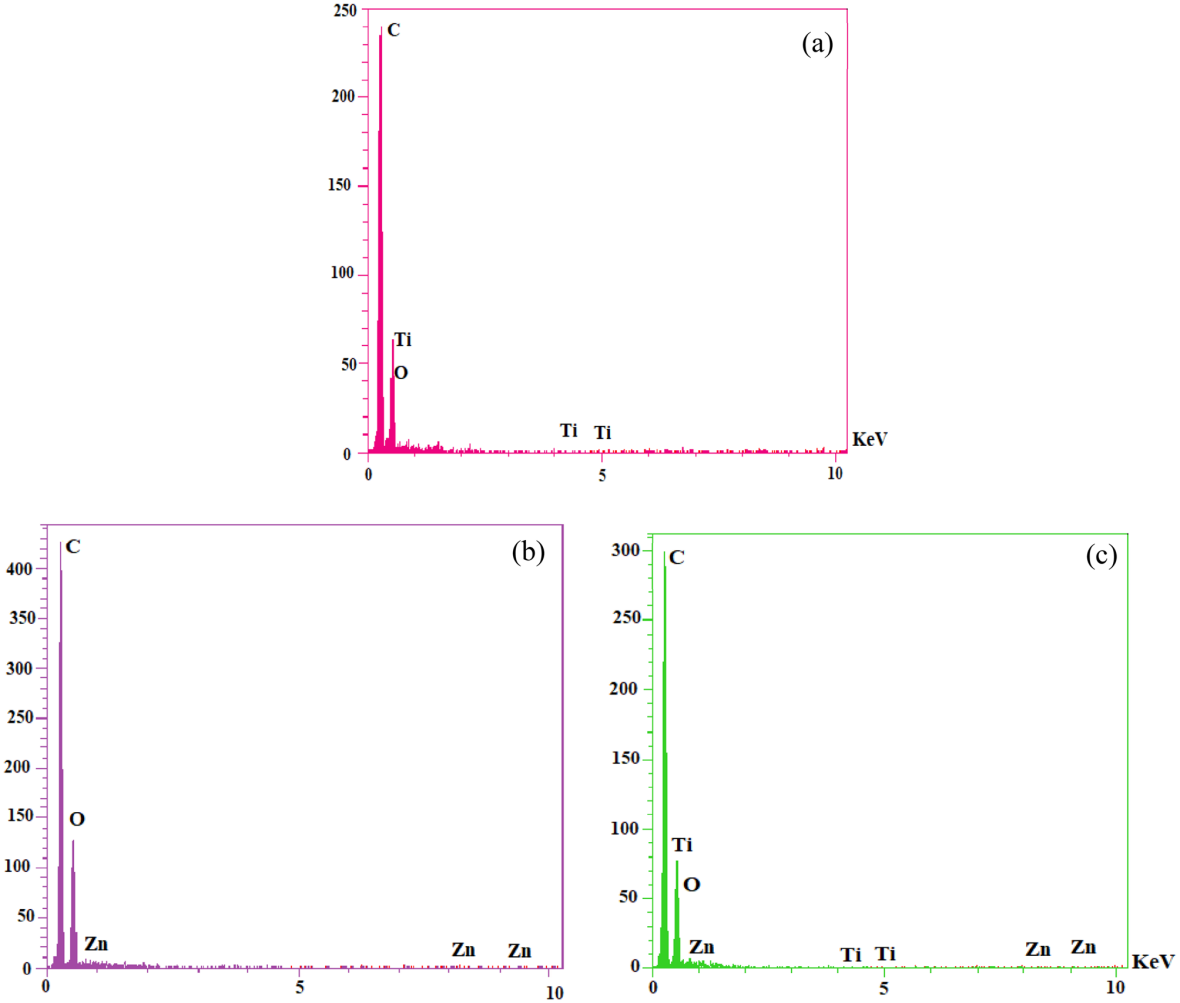
EDX analyses of (**a**) PMMA/TiO_2_, (**b**) PMMA/ZnO, and (**c**) PMMA/TiO_2_:ZnO nanohybrid coatings.

To determine the chemical composition of the prepared nanocoatings, the EDX analysis was carried out, as shown in Fig. 16. Figure 16a depicts the EDX spectra of PMMA + 0.1 wt% TiO_2_ nanoparticles. According to the EDX, the presence of C, Ti, and O elements in the film structure was proved. Remarkably, the presence of carbon elements in all spectra is due to the presence of CH_3_ and CH_2_ groups in the structure of PMMA coating, as clearly explained in the FTIR section. Figure 16b demonstrates the EDX spectra of PMMA + 0.1 wt% ZnO nanoparticles. It can be noticed that the presence of zinc along with carbon and oxygen in the aforementioned nanocoating film. In addition, Fig. 16c depicts the EDX spectra of PMMA + 0.05 wt% TiO_2_: ZnO hybrid. The spectrum shows the elemental composition of Ti, Zn, C, and O.

## Conclusion

This study aimed to prepare a UV-shielding coating thin film based on the PMMA matrix. On this basis, PMMA granules were solved in tetrahydrofuran and TiO_2_, ZnO, and TiO_2_: ZnO hybrid as nanofillers were added at different contents of 0.05, 0.1, and 0.2 wt%. To obtain the optimal ratio of nanohybrids, the nanohybrid coatings were prepared at different ratios of nanoparticles, and 50%:50% showed the best features. The prepared samples were in contact with a UV lamp for 720 h to study the degradation effect. According to the FTIR and UV–Vis analyses outcomes, the obtained nanocoating films exhibited excellent UV-shielding performance. PMMA coating with 0.1 wt% TiO_2_, 0.1 wt% ZnO, and 0.05 wt% TiO_2_: ZnO hybrid could improve the transparency of the prepared coating better than other samples. Furthermore, XRD patterns revealed that adding nanoparticles to the pure film did not change the amorphous nature of the polymer. SEM micrographs and histograms confirmed that the utilized TiO_2_ and ZnO nanoparticles had a spherical shape which were prone to be agglomerated with an average diameter size of 42.5 nm and 13.5 nm, respectively. It can be deduced that by adding a proper amount of nanoparticles to the polymer host film, the resistance property of the film could be improved when exposed to UV rays.

## Data Availability

All data generated or analysed during this study are included in this published article.
